# Factors affecting utilization of antenatal care in Ethiopia: A systematic review and meta-analysis

**DOI:** 10.1371/journal.pone.0214848

**Published:** 2019-04-11

**Authors:** Tesfalidet Tekelab, Catherine Chojenta, Roger Smith, Deborah Loxton

**Affiliations:** 1 Research Centre for Generational Health and Ageing, School of Medicine and Public Health, University of Newcastle, Newcastle, New South Wales, Australia; 2 College of Medical and Health sciences, Wollega University, Nekemte, Oromia region, Ethiopia; 3 The Mothers and Babies Research Centre, School of Medicine and Public Health, University of Newcastle, Newcastle, New South Wales, Australia; The University of Adelaide School of Agriculture Food and Wine, AUSTRALIA

## Abstract

**Background:**

In the context of high maternal morbidity and mortality in Sub-Saharan Africa, less than 80% of pregnant women receive antenatal care services. According to a 2016 national report, only 62% of pregnant women in Ethiopia made at least one antenatal care visit. The aim of this review was to systematically and quantitatively summarize the factors affecting utilization of antenatal care in Ethiopia.

**Methods:**

We searched PubMed, Medline, EMBASE, CINAHL, Google Scholar and Maternity and Infant Care database for studies that had been conducted in Ethiopia between 2002 and 2016. We summarized the studies on the use of antenatal care services quantitatively and qualitatively. A random-effects model was conducted to obtain the pooled estimates.

**Results:**

A total of fifteen observational studies were included in this review. The pooled prevalence of utilization of antenatal care services in Ethiopia was 63.77% (95CI 53.84–75.54). The pooled odds ratio showed that a significant positive association was found between utilization of antenatal care and urban residence (OR = 1.92, 95%CI = 1.35–2.72), women’s education (OR = 1.90, 95%CI = 1.52–2.37), husband’s education (OR = 1.49, 95%CI = 1.32–1.69) and planned pregnancy (OR = 2.08, 95%CI = 1.45–2.98). Based on narrative synthesis exposure to mass media, family income and accessibility of the service were strongly associated with utilization of antenatal care.

**Conclusion:**

The findings of this review found several modifiable factors such as empowering women through education and increasing their decision-making power, promoting family planning to prevent unplanned pregnancy, increasing awareness of women through mass media and making services more accessible would likely to increase utilization of antenatal care. Further research is needed on accessibility and availability of the service at the individual and community level to assess the predictors of antenatal care service utilization.

## Introduction

Pregnancy is an important time to promote good health and prepare women and their families psychologically and emotionally for parenthood. Antenatal care (ANC) can be defined as the care provided by skilled health-care providers to pregnant women and adolescent girls in order to ensure the best health conditions for both mother and baby during pregnancy [1]. Antenatal care is one of the “four pillars” of safe motherhood initiatives to promote and establish good health during pregnancy and the early postpartum period [2, 3]. Good quality antenatal care services improve the survival and health of mothers as well as babies. Antenatal care also provides an opportunity for women to communicate with their healthcare provider and increases the chances of their using a skilled birth attendant [2, 4].

Previously the World Health Organization (WHO) recommended four antenatal visits for uncomplicated pregnancies. The first of these took place within 12 weeks of gestational age [5]. However, in a recently published document, WHO now recommends a minimum of eight visits to improve neonatal outcomes and to provide a more positive and women-centred experience for clients [1]. In sub-Saharan Africa, improving maternal and newborn health remains a major challenge [6, 7]. Most maternal and newborn deaths and pregnancy-related complications can be prevented, detected and managed if pregnant women receive quality maternal healthcare services from a skilled health worker [8–11]. In developing countries, where maternal mortality is fourteen times higher than in high income countries, only 52% of pregnant women received the recommended number of antenatal care visits in 2014 [12]. In Sub-Saharan Africa, about 80% of pregnant women attended at least one antenatal care visit and 52% of pregnant women received the (then) recommended number of four antenatal care visits in 2016 [13]. Antenatal care may not prevent all causes of maternal and newborn deaths, but it may facilitate the early detection and prevention of many existing diseases [14, 15].

In Ethiopia, as in other low-income countries, there are high rates of maternal and newborn death, and the utilization of antenatal care is low [16]. The high mortality rate in Ethiopia is reflective of the low utilization of maternal healthcare services [17]. According to the 2016 Ethiopian Demography and Health Survey (EDHS), antenatal care service utilization was 62% and only 20% of women had their first antenatal care during the first trimester. Only 32% of women had four antenatal care visits during their pregnancy [18].

In previous studies, it has been reported that utilization of antenatal care is influenced by a range of factors such as individual level (socio-economic and reproductive characteristics), household level or interpersonal level (women’s autonomy, husband attitude and support, family income) and health service level (distance, accessibility and availability) [19–24]. In a recent review conducted in low and middle-income countries, Banke-Thomas OE stated that education of the mother and her partner were the most significant factors that influence the utilization of maternal healthcare services [25]. In a study carried out in sub-Saharan Africa countries, it was revealed that use of antenatal care was associated with mother’s age, parity, interaction with healthcare provider and cost of antenatal care etc.[21]. The findings of a systematic review conducted by Simkhada and colleagues, indicated that womens’ and their partners’ level of education and exposure to mass media were associated with the utilization of antenatal care [24]. Results of a recent study carried out in Sudan indicated that the uptake of antenatal care services was higher for mothers with high educational attainment (secondary education and above) (94.1%) compared to mothers with no education (67.1%) [26]. Observational studies conducted in Ethiopia indicated that education, residence, mass media, marital status were associated with the utilization of antenatal care [27–30].

While several studies focusing on determinants of antenatal care use in Ethiopia have been published, they provide mixed results or identify several determinants as important. This review is therefore necessary to obtain an overall picture of which determinants are important and how much of an impact they have on antenatal care use. This information necessary for policy planners and program managers to identify gaps in the utilization of antenatal care, and to plan strategies to increase the utilization of services. Moreover, no other studies examined comprehensively about utilization of antenatal care in Ethiopia. The aim of this review is to therefore systematically and quantitatively summarize the factors affecting the utilization of antenatal care among women in Ethiopia who were pregnant or had given birth at least once preceding the survey.

## Methods

### Study selection

The Preferred Reporting Items for Systematic Reviews and Meta-Analyses (PRISMA) checklist was used in the formulation of the systematic review methodology [31]. The systematic review was registered on the PROSPERO prospective register of systematic reviews (registration number: CRD42016045866).

Articles identified through the search in the below listed electronic databases were assessed for relevance by first screening titles and then abstracts, where necessary. Studies identified as meeting the eligibility criteria from the title and abstract screening process then underwent full text review. A review of reference lists of the retrieved articles was also conducted to assess any other relevant additional articles that may have been missed in the search.

### Criteria for inclusion of studies

#### Study design and period

Observational studies reporting factors affecting the utilization of antenatal care in Ethiopia published between 2002 and 2016 were considered. The year 2002 was selected as the starting point as focused antenatal care was not available in Ethiopia prior to this. Only English-language full-text reports were included.

**Study setting**: Community-based studies considering women in the reproductive age groups.

**Participants**: Women who were pregnant or had given birth at least once preceding the survey.

**Exposure**: Predictors/determinants of antenatal care. The determinants are characteristics or exposure that increase or decrease the likelihood of antenatal care use. These may be related to educational status, residence, maternal age, parity, marital status etc.

**Outcome**: Pregnant women having at least one antenatal care visit.

### Search strategy

All studies published between 2002 and 2016 were systematically searched through electronic databases including PubMed, Medline, EMBASE, CINAHL, Google Scholar and Maternity and Infant Care databases(S1 Table). Searches was conducted using terms such as “antenatal care” or “prenatal care”, or “maternal health care”, “antenatal care” and “utilization” and “Ethio?”, “prenatal care” and “utilization” and “Ethio?” “factors”, “maternal health care” and “utilization” and “Ethiopia”, “antenatal care” or “prenatal care” or “maternal health care” and “factors” and “Ethiopia”, “antenatal care” or “prenatal care” or “maternal health care” and “determinant factors” and “Ethiopia”, “antenatal care” or “prenatal care” or “maternal health care” and “Ethio?”.

### Data extraction

Data were extracted from articles included in the review using a data extraction tool which was developed by our team with clear inclusion and exclusion criteria. All authors developed this data extraction sheet, confirming that it would adequately capture data required to answer the review questions. Two authors (TT & CC) extracted data from the included studies. For each study included, we recorded the last name of author(s), year of publication, exposure measurement (women’s age, women’s education, partner’s education, residence, parity, marital status, type of pregnancy etc.), the response, study methods (study setting, study participants, study design, the year of data collection, sample size and data analysis), study region, odds ratio of antenatal care use with 95% confidence interval and percentage of antenatal care utilization. When clarification was required, we contacted the primary authors of the studies to resolve any uncertainties.

### Risk of bias (quality) assessment

All articles selected for inclusion in the review were assessed rigorously by review authors (TT &CC). To measure the risk of bias within included studies, the methodological quality of potential studies was assessed by using the Newcastle-Ottawa scale (NOS) for assessing the quality of observational studies in systematic reviews and meta-analyses [32].

Selection of study groups was assessed by looking at sample representativeness, ascertainment of exposures, sample size and non-response rate; comparability was assessed by looking at the comparability of the subject and outcome was assessed based on assessment of the outcome and statistical test for cross-sectional studies. For cohort studies, selection of study groups was assessed by looking at the representativeness of the exposed cohort, selection of the non-exposed cohort, ascertainment of exposure and demonstration that outcome of interest was not present at the start of study. Comparability was assessed by comparability of cohorts on the basis of the design or analysis, and outcome was assessed based on assessment of outcome, whether follow-up was long enough for outcomes to occur and adequacy of follow up of cohorts. Based on NOS, studies were awarded a maximum of four stars in the selection category, two-stars in comparability and three stars within outcome. In this systematic review and meta-analysis, studies with less than seven stars were considered low quality, and those with seven stars or more were considered high quality. Any disagreements that arose between reviewers were resolved through discussion between the reviewers, or with a third reviewer.

### Data synthesis and analysis

The data entry and statistical analysis was carried out in Comprehensive Meta-Analysis (CMA) V2 software. Tables and figures were used to summarize the selected studies and results descriptively. We also implemented a meta-analysis of studies that provided a comparable classification of the determinants or exposures and the outcome variables. For the purpose of meta-analysis, we considered estimates of adjusted odds ratio with the confidence interval (CI) as the measure of association. The overall effect (pooled estimates of the magnitude and the factors) of antenatal care service was estimated using a random effect model and measured by the prevalence rates and odds ratio with 95% CI. We selected the random effect model because of heterogeneity due to difference in the study design and study regions. To determine heterogeneity among studies, we calculated the I^2^ statistic, which describes the percentage of total variation among studies due to heterogeneity rather than to chance. An I^2^ statistic value of 30% to 60%: may represent moderate heterogeneity; 50% to 90%: may represent substantial heterogeneity; 75% to 100%: considerable heterogeneity [33, 34]. Furthermore, sensitivity analysis was conducted to assess the stability or robustness of the pooled estimates to outliers and the impact of individual studies. Due to heterogeneity among studies, we performed sub-group analysis based on study design, region and quality of studies.

In order to check publication bias, funnel plot asymmetry and the Egger’s test of the intercept in random effects model was used [35].

### Operational definitions

**Antenatal care service utilization-** women having at least one antenatal care visit.

**Marital status**- classified as married and other(including divorced, single and widowed). The second used as the reference category.

**Residence-** grouped as rural and urban. Rural indicated as a reference group.

**Parity**- classified as women’s having one to four living children and more than four children. The latter used as reference point.

**Type of pregnancy**- women were assigned to type of pregnancy (planned versus unplanned). The second used as the reference category.

**Age of the mother**- Women were assigned to age category (< = 19 years or >19 years). The second used as the reference category.

**Women’s Educational status**—Women were assigned an educational status (No education/educated). The first used as the reference category.

**Husband/Partner education**- women whose husbands educated/no educated. The second used as the reference category.

## Results

### Search results

**Fig 1:**
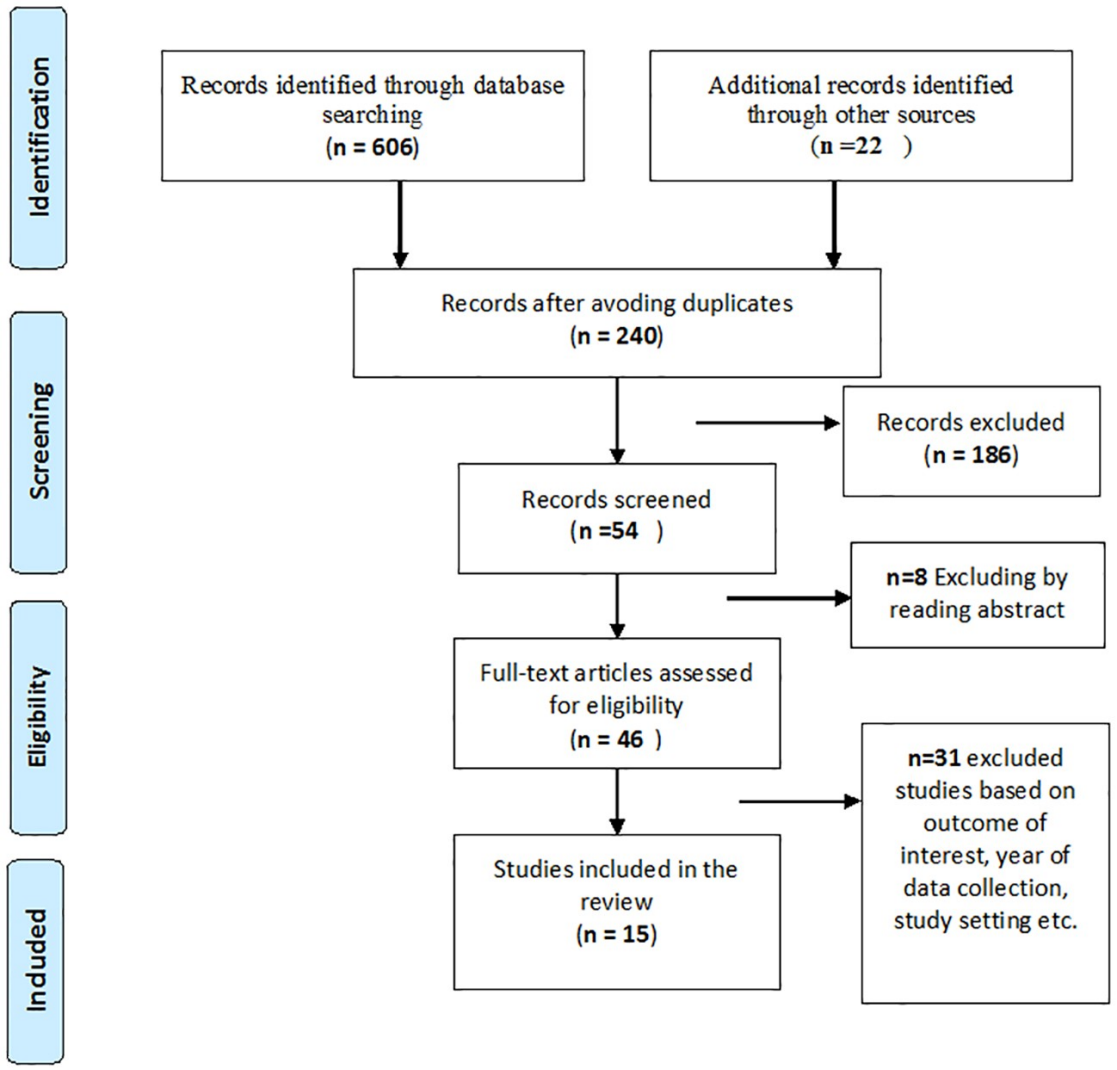
Flow chart diagram describing selection of studies included in the systematic review and meta-analysis using PRISMA checklist. Studies may have been excluded for more than one reason.

We conducted our search between 18^th^ July and 27^th^ August 2016 and identified 628 studies, as shown in (Fig 1). After removing duplicates, 240 records remained. Titles and abstracts of retrieved articles were assessed. After applying the inclusion and exclusion criteria, 15 studies remained.

### Study characteristics

Characteristics of the included studies are presented in Table 1. The majority of included studies employed a cross-sectional design (n = 13); two cohort studies were also included. A total of 20,185 women from fifteen studies were included in this systematic review and meta-analysis. The sample size ranged from 307 to 7,908 women. Five studies were conducted in SNNPR, four studies in Oromia region, two in Benishangul Gumuz region, two studies in Tigray, one in Amhara region and one was a nationally based study. The majority of participants were women who had given birth in the past five years, and the participants for two studies were pregnant women in their third trimester. The magnitude of antenatal care service utilization ranged from 28.5% to 90.6%.

**Table 1:** Summary of characteristics of included studies.

First Author and Year	Study design	Region	Year of data collection	Study Population	Sample size	Response	Exposure Measurement	Outcome measurement (One antenatal care visit)	ANC utilization
Birmeta K et al, 2013	CS	Oromia	January to Febraury,2012	Women who had given birth in the past three years	422	99.2%	Age at last birth, Parity, Literacy status, Occupation, Marital status, Income, Media exposure, Type of pregnancy, Knowledge on danger signs of pregnancy, Presence of husband approval on ANC	Maternal health care (Antenatal care and skilled delivery) Antenatal care–women who received ANC at least once	87.1%
Abosse Z et al, 2010	CS	SNNPR	January to February,2009	Women who had given birth in last 5 years interviewed	710	97.3%	Age, Residence, Ethnicity, Occupation, Religion, Marital status, Family size, Income, Educational status, Positive husband attitude, Parity, Ever had abortion, Planned pregnancy, Belief about risk of pregnancy, distance	Antenatal care service—women who received ANC at least once	86.3%
Amentie MA et al, 2015	CS	Benishangul Gumuz	May17-31,2012	Women who had given birth in past five years	536	97.9%	Place of residence, Religion, Ethnicity, Educational status, Availability of traditional birth attendants kebele, Awareness on ANC service, Transportation and distance	Antenatal care services—women who received ANC at least once	81.9%
Melaku, YA et al. A. 2014	Longitudinal cohort study	Tigray	September,2009 –August,2013	Pregnant women and women who had given birth between September 2009 and August 2013	2361	98.2%	Age, Residence, Maternal Educational status, Marital status, maternal occupation	Antenatal care and institutional delivery. Antenatal care—women who received ANC at least once	76.5%
Dutamo, Z et al, 2015	CS	SNNPR	January 1–31,2014	Women who had given birth in the last year	634	98.2%	Age, Mother’s education, Husband education, Employment status, Women’s autonomy, Monthly income, parity, Pregnancy intention, Aware danger signs of pregnancy	Maternal Health Care (Antenatal care and skilled birth delivery). Antenatal care—women who received ANC at least once	87.6%
Jira C & Belachew T, 2005	CS	Oromia	February 1–20,2004	Pregnant women in their third trimester	307	100%	Age, marital status, Occupation, religion, income, pregnancy intention, Husband attitude towards ANC, Women’s awareness on ANC utilization	Antenatal care- women who received ANC at least once	90.6%
Girmay M & Berhane Y,2016	CS	SNNPR	January to February,2015	Women who had given birth in one year preceding the study	778	96.1%	Residence, Maternal Education, Husband education, ANC follow up for previous pregnancy, Complications during previous pregnancies or births, Skilled personnel preferred for ANC services, Awareness on places to get skilled providers for ANC, Listen to radio, Main road to nearest health facilities	Skilled antenatal care services—women who received ANC at least once	71%
Regassa N,2011	CS	SNNPR	2011	Women who had a child less than 24 months	1,094	100%	Age of women, Children ever born, Religion, Radio listening frequency, Pregnancy intention, Employment, Women’s literacy status	Antenatal and postnatal care. Antenatal care—women who received ANC at least once	77.4%
Tarekegn SM et al, 2014	CS	National	December 27,2010 –June,03,2011	Women who had at least one birth within the last 5 years	7,908	100%	Residence, Marital status, Age, religion, Ethnicity, Educational status, Wealth, Parity, number of births, Husbands education, Women’s autonomy, Husband’s work status, Women’s work status, Reading newspaper frequency, Listening radio frequency, Watching television frequency	Maternal Health Service (Antenatal care and skilled delivery). Antenatal care- women who received ANC at least once	33.9%
Tewodros B et al, 2009	CS	SNNPR	April,2008	Women who had given birth in the past twelve months	651	96.3%	Residence, distance, Presence of Husband Approval, Age, Did you plan your last pregnancy, Educational Status of women, Know danger signs of pregnancy, Exposure to Illness in past pregnancies, Perceived Susceptibility in future pregnancies	Antenatal care—women who received ANC at least once	28.5%
Tsegaye Y et al, 2013	CS	Tigray	August–September, 2009	Women who had given birth at least once in the five years	1,115	99%	Age, marital status, Education, Parity, Health facility in village, Husbands Occupation	Antenatal and delivery care. Antenatal care- women who received ANC at least once	54%
Tura G,2009	CS	Benishangul Gumuz	January,25—February,10,2007	Women who had at least one delivery in the past five years	1060	97.9%	Place of residence, educational status, Occupation, Husband’s education, Husband’s occupation, Have radio, Monthly income, Knowledge on ANC	Antenatal care—women who received ANC at least once	49.5%
Worku AG et al, 2013	Population based cohort study	Amhara	January–March,2012	Women who had births in the year preceding the survey	1730	96.4%	Mother’s education, Husband education, Wealth quintile, Awareness on risk of pregnancy, Awareness on places to get skilled provider, Birth order, Pregnancy wontedness, ANC in previous pregnancy, Income, Average distance to the nearest health centre	Skilled maternal care (Antenatal care, Skilled delivery and postnatal care) Antenatal care- women who received ANC at least once	32.3%
Zelalem AD et al, 2014	CS	Oromia	June,2012	Women who gave at least one live birth in the five years	495	100%	Age, maternal education, family size, Health education on maternal health, History of abortion, Means of transport, Perception of quality of services, residence	Maternal Health care(Antenatal care and institutional delivery) Antenatal care- women who received ANC at least once	86.1%
Fekede B & Gebremariam A,2007	CS	Oromia	Januray,26 –February 06,2006	Pregnant women in their third trimester	384	93.8%	Age, marital status, Occupation, Monthly income, Ethnicity, religion, gravidity,	Antenatal care service utilization- women who received ANC at least once	76.7%

As shown in Table 2; of the 15 studies, five studies were deemed low quality. The shortfalls of the low-quality studies included were lack of representativeness [36–39], the sample size was not justified [39] and statistical method not described clearly [39, 40].

**Table 2:** Quality assessment of included studies based on NOS checklist.

Study & Ref	Selection (Maximum of four star)	Comparability (maximum two star)	Outcome assessment (Maximum of three stars)	Quality	Exposures (Meta-analysed)
Birmeta, K et al	****	**	***	High	**Maternal literacy**- No schooling- R, Schooling- 2.65(1.08–6.49) **Age at last birth**—15-19-2.04(0.33–12.74), > = 20- R, **Marital status**—Married—1.07 (0.40–2.84), Others (divorced, widowed, never married)- R, **Parity** 1-4-0.81(0.32–2.06), >5 –R, **Pregnancy intention**- Yes- 2.94(1.15–7.53), No–R
Abosse, Z et al	**	**	**	Low	**Maternal education**—No education—R, Primary school and above- 0.68(0.13–3.58); **Residence**- Urban-0.39(0.13–1.17), Rural-R
Amentie M et al	***	**	**	High	**Educational status**- Illiterate–R, Literate 3.24(1.84–5.72), **Place of residence-** Urban -3.70(0.83–16.43), Rural-R
Melaku, Y. A et al	***	**	****	High	**Maternal educational status**—No education -R, Educated -1.62(1.25–2.10), **Residence**- Urban -2.20(1.25–3.87), Rural-R; **Age (Years)**– 15-19-0.50(0.40–0.63), > = 20-R, **Marital status**- Married-1.45(0.87–2.42), Others (Single, dissolved)- R
Dutamo, Z et al	***	**	**	High	**Mother’s education**—No education- R, Primary and above -1.68(0.96–2.94), **Husband education—**No education- R, Primary and above -1.52(0.88–2.62), **Age group of women**- 15-19- **1.1** (0.30, 4.06), > = 20-R; **Parity**- Parity 1–4 2.62(1.56–4.40), Parity >4 –R; **Pregnancy intention**- Intended -1.90(1.01–3.59), Unintended–R
Jira C and Belachew T	**	*	**	Low	**Pregnancy intention**–Yes-1.18(0.31–4.52), No-R
Girmay M & Berhane Y	***	**	**	High	**Maternal education**—Education-1.32(0.49–3.58), No education-R, **Residence**- Urban- 1.01(0.04–27.06), Rural-R, **Husband education**-Education- 1.61 (0.60, 4.35), No education- R,
Regassa N	***	*	**	Low	**Women’s literacy status**- Literate-1.39(1.01–1.92), Illiterate–R, **Pregnancy reaction**- Wanted-2.17(1.56–3.02), Unwanted-R
Tarekegn SM et al	****	**	**	High	**Educational status**- No education–R, Primary and above—2.39(1.72–3.33), **Residence**- Urban 2.3(1.81–2.92), Rural-R, **Husband education**- No education–R, Primary and above -1.60(1.36–1.88); **Age**- 15-19- 0.80(0.60, 1.28), > = 20-R, **Marital status**- Others(Never married, Divorced/separated/widowed)- R, Married-0.9(0.55–1.46), **Parity–** 1–4–0.83(0.66–1.04), > = 5 –R
Tewodros B et al	***	**	**	High	**Educational Status of women**- Illiterate–R, Primary and above-3.90(2.27–6.71), **Residence**- Urban-2.11(1.01–4.42), Rural-R, P**regnancy**- Planned-4.14(2.18–7.86), Unplanned-R
Tsegaye Y et al	***	**	***	High	**Education**- No Education-R, Primary school and above- 1.45(1.05–2.00); **Marital status**- Others(Single or widowed, Divorced)–R, Married-2.57(1.44–4.58), **Parity**- 1-4-R, 5-7-1.16(0.88–1.55), 8-11-1.28(0.87–1.88)
Tura G	***	*	**	Low	**Place of residence**- Urban- 1.60(0.99–2.58), Rural- R; **Educational status**- No education- R, Educated– 6.25(1.49–26.27)
Worku AG et al	***	**	***	High	**Mother’s education**- No education R, Primary and above- 1.26(0.98–1.62);, **Husband education**—No education R, Primary above- 1.28(1.03–1.60), **Pregnancy wontedness**- Wanted-1.27(0.82–1.96), Unwanted-R
Zelalem, AD et al	***	*	***	High	**Educ. Respondent**- Illiterate (RC)-R, Primary and above- 2.59(1.09–6.15); **Residence**—Urban -5.46(1.13–26.29), Rural-R,
Fekede B & Gebremariam A	**	*	**	Low	**Age in years**—15-19- 2.74(1.38–5.43), > = 20-R, **Marital status**—Others(Single or widowed, Divorced)–R, Married-0.74(0.42–1.31, **Parity**: 1-4-1.75(0.50–6.15), >4-R

### Magnitude of antenatal care service utilization

**Fig 2:**
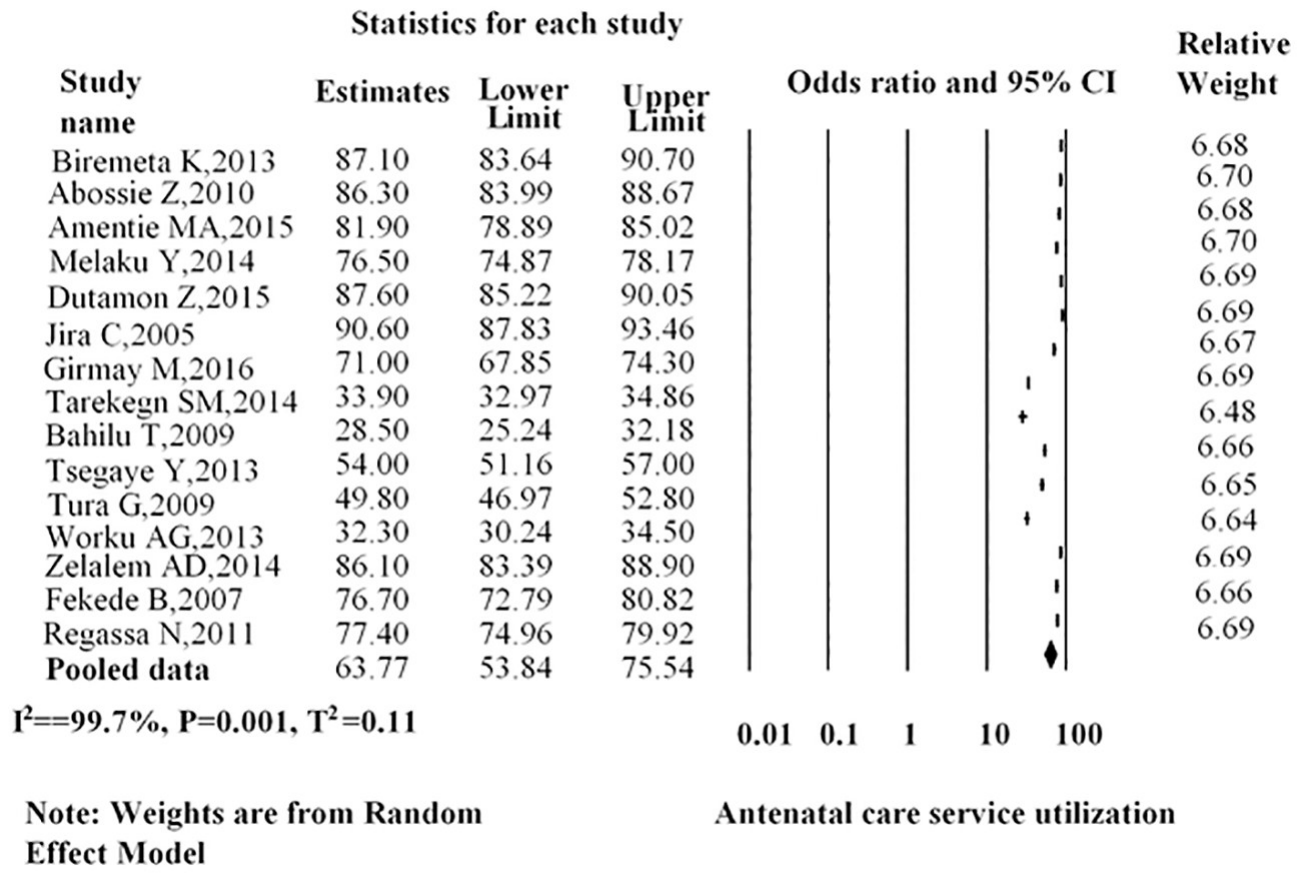
Overall pooled estimates of antenatal care service utilization in Ethiopia, 2016.

As shown in (Fig 2), the overall point estimate of antenatal care among women in Ethiopia was 63.77% (95%CI: 53.84–75.54).

The subgroup analyses showed that the overall magnitude of antenatal care among women in Ethiopia based on study design was 65.33% (95%CI: 54.28–78.63), which is 66.24% (95%CI: 54.78–80.09) for the cross-sectional studies and 49.74% (95%CI: 21.37–115.78) for the cohort study. Subgroup meta-analysis of the prevalence of antenatal care by region showed a higher pooled estimate of antenatal care in Oromia of 85.21% (95%CI: 80.36–90.34) and SNNPR region, 66.40% (95%CI: 56.68–77.77). We also performed a sub-group analysis based on the quality of studies, and the overall estimates revealed that the magnitude of antenatal care in Ethiopia was 69.84% (95%CI: 61.39–79.45) which was 58.90% (95%CI: 46.25–66.00) for high quality studies and 74.73% (95%CI: 64.17–87.03) for low quality studies (see Table 3).

**Table 3:** Sub-group analysis of studies included in meta-analysis on factors affecting utilization of antenatal care in Ethiopia.

Sub-group	Random effects (95%CI)	Test of heterogeneity I^2^
**Study design**		
Prospective cohort	49.74(21.37–115.78)	99.8
Cross-sectional	66.24(54.78–80.09)	99.7
Over all	65.33(54.28–78.63)	99.7
**Quality**		
High	58.90(46.25–66.0)	99.7
Low	74.73(64.17–87.03)	98.8
Over all	69.84 (61.39–79.45)	99.7
**Region**		
Oromia	85.21(80.36–90.34)	89.7
Tigray	64.33(45.73–90.50)	99.2
Benshangul Gumuz	63.90(39.24–104.04)	99.5
SNNPR	66.40(56.68–77.77)	98.9
Amhara	32.30(30.24–34.50)	-
National	33.90(32.97–34.86)	-
Overall	39.69(38.78–40.62)	99.7

To assess the robustness of the magnitude of antenatal care results, we carried out a leave-one-out sensitivity analysis by repeating and removing one study at a time and recalculating the summary of the effect size. The summary effect size remained constant, showing that our results were not determined by any single study.

### Determinants of antenatal care service utilization

In this review, some of the factors associated with the utilization of antenatal care were pooled quantitatively and some were not because of inconsistent classification (grouping) of the exposures with respect to the outcome (antenatal care service utilization).

**Fig 3:**
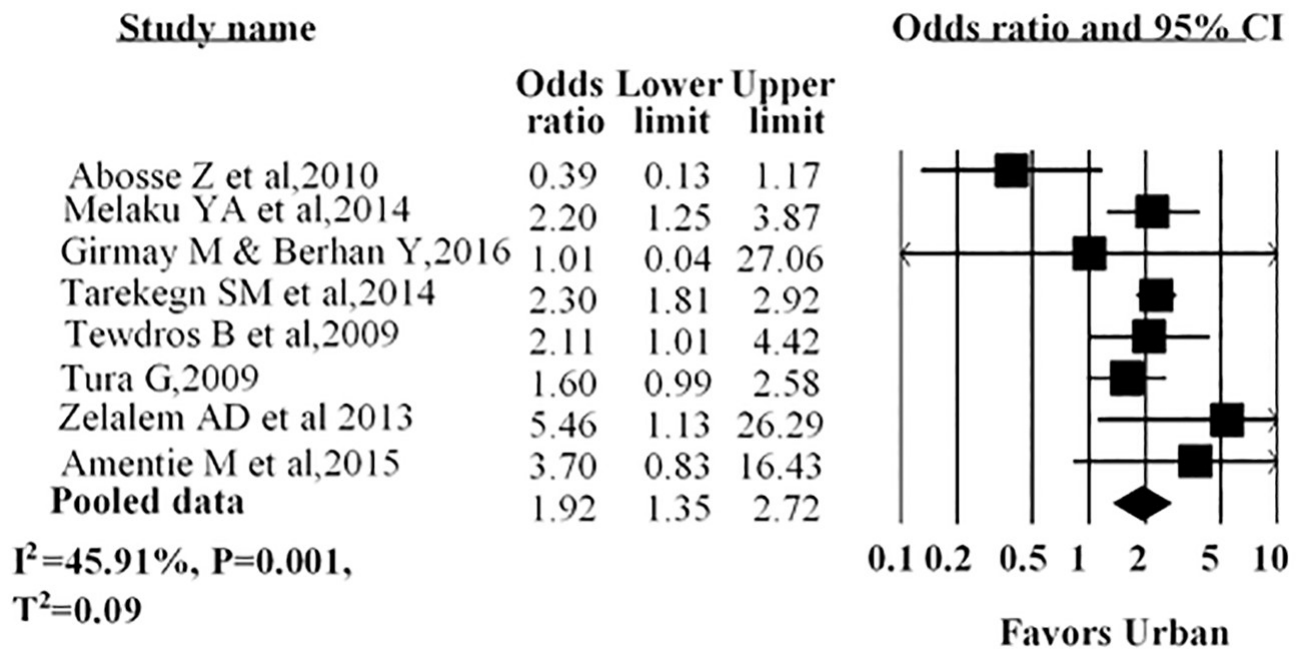
Forest plot for the association between residence and use of antenatal care service in Ethiopia, 2016. Notes: In each study, women were assigned to an area of residence (urban/rural). Each adjusted odds ratio is an estimate for a comparison between the women in urban residence and those in rural, with the latter used as the reference category.

Four studies indicated that women residing in urban areas were more likely to receive antenatal care service utilization. The remaining four studies reported that place of residence had no association with antenatal care service. Our pooled data indicated that women who resided in urban areas were nearly two times more likely to utilize antenatal care than their rural counterparts (AOR = 1.92; 95%CI: 1.35–2.72) (Fig 3).

**Fig 4:**
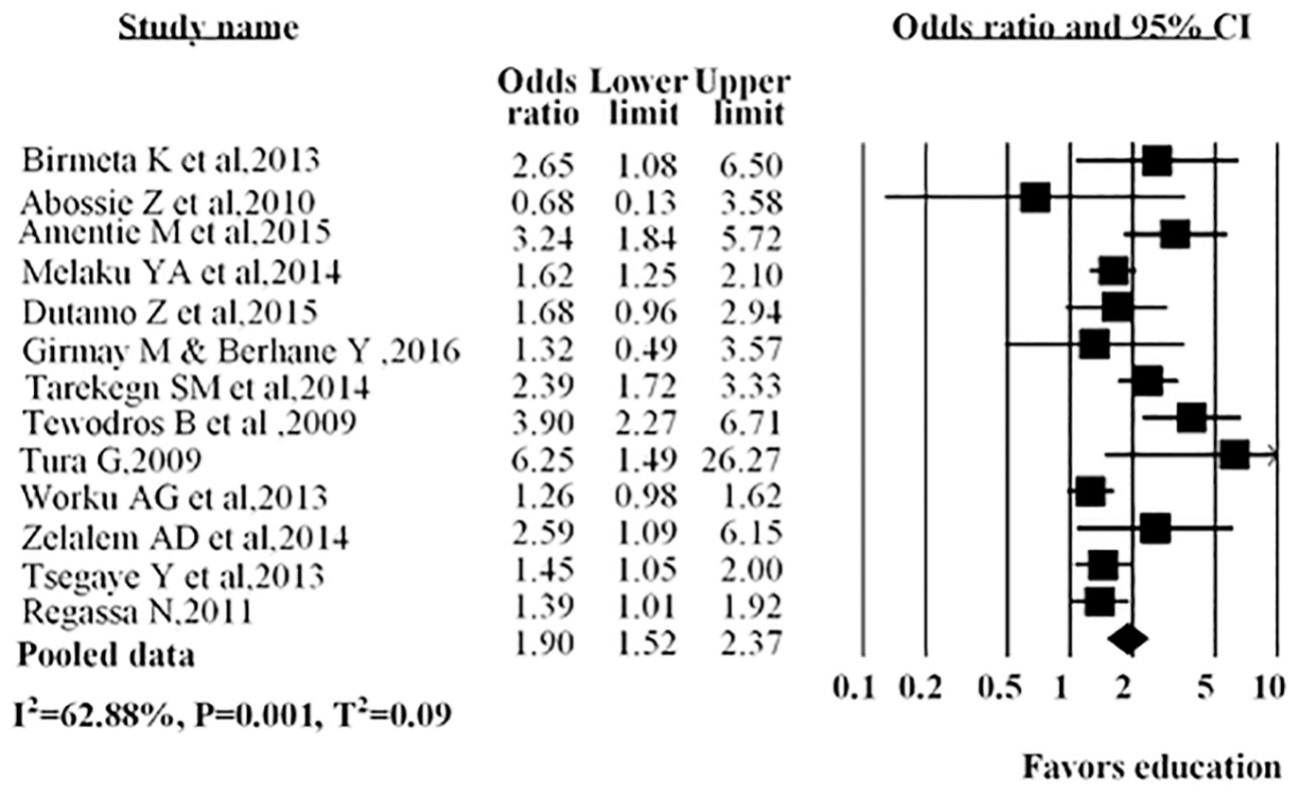
Forest plot for the association between educated women and use of antenatal care service in Ethiopia, 2016. Notes: In each study, women were assigned to educational status (No education/educated). Each adjusted odds ratio is an estimate for a comparison between the women with no education and educated, with the first used as the reference category.

Education was assessed in 13 studies, in nine studies it was shown that educated women were more likely to use antenatal care service than uneducated women. The overall estimates revealed that educated women (AOR = 1.90; 95%CI: 1.52–2.37) were more likely to use antenatal care than those without education (Fig 4).

**Fig 5:**
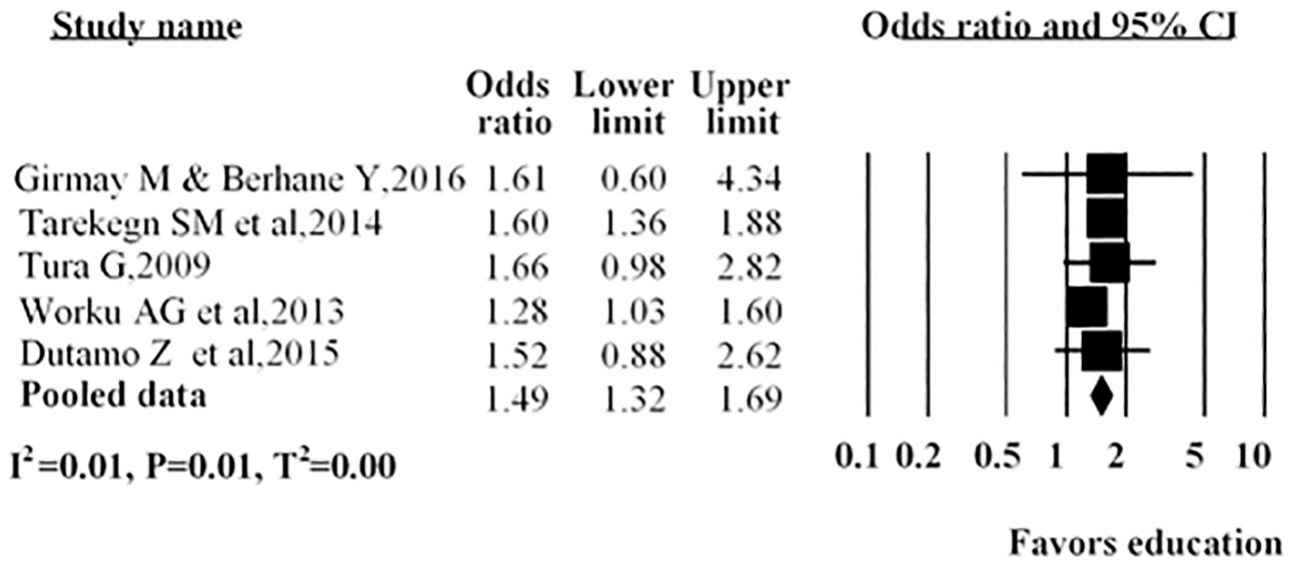
Forest plot of five studies included in a meta-analysis assessing the association between husband or partner education and utilization of antenatal care in Ethiopia, 2016. Notes: In each study, women were assigned based on husband educational status (No education/educated). Each adjusted odds ratio is an estimate for a comparison between no education and educated, with the first used as the reference category.

As shown in (Fig 5), several studies described the husband’s or partner’s education; three reported no association between the husband’s or partner’s education with antenatal care service utilization, while in the other two studies a positive association was found with antenatal care service utilization. The overall pooled estimate indicated that antenatal care service utilization more likely with those women whose husbands were educated compared to their counterparts (AOR = 1.49; 95%CI: 1.32–1.69).

**Fig 6:**
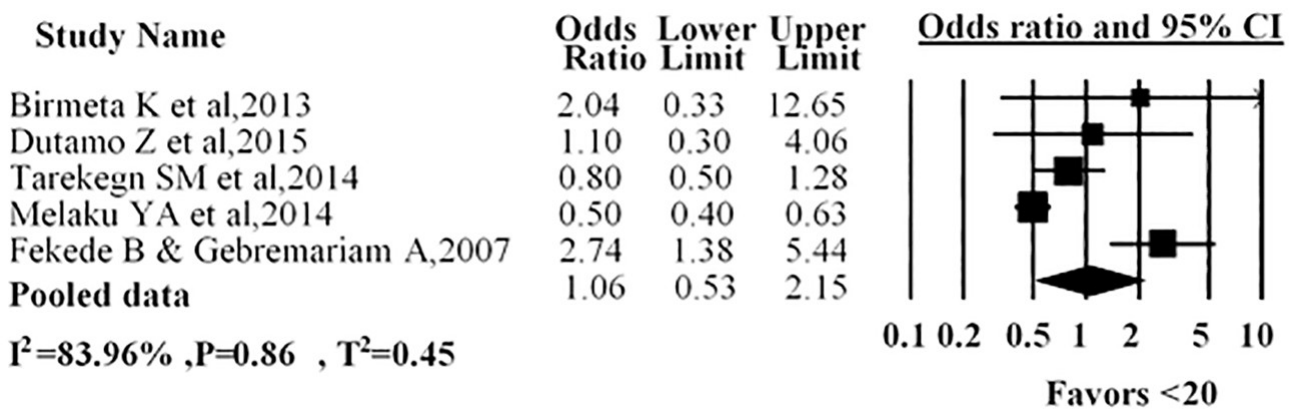
Forest plot for the association between age of the women and use of antenatal care service in Ethiopia, 2016. Notes: In each study, women were assigned to age category (l<20 years />=20 years). Each adjusted odds ratio is an estimate for a comparison between the women with age group <20years and >+20years, with the second used as the reference category.

Five studies presented the mother’s age with antenatal care service utilization; in three studies it was indicated that women in the age group of less than twenty years had no association with antenatal care service utilization. One study indicated that women younger 20 years were less likely to utilize antenatal care than those over 20 years. Another study revealed that women in the age group of less than twenty years more likely to use antenatal care than women in the age group twenty and above. Our pooled estimates showed no association with a younger age (AOR = 1.06; 95%CI: 0.53–2.15) and antenatal care service utilization in Ethiopia (Fig 6).

**Fig 7:**
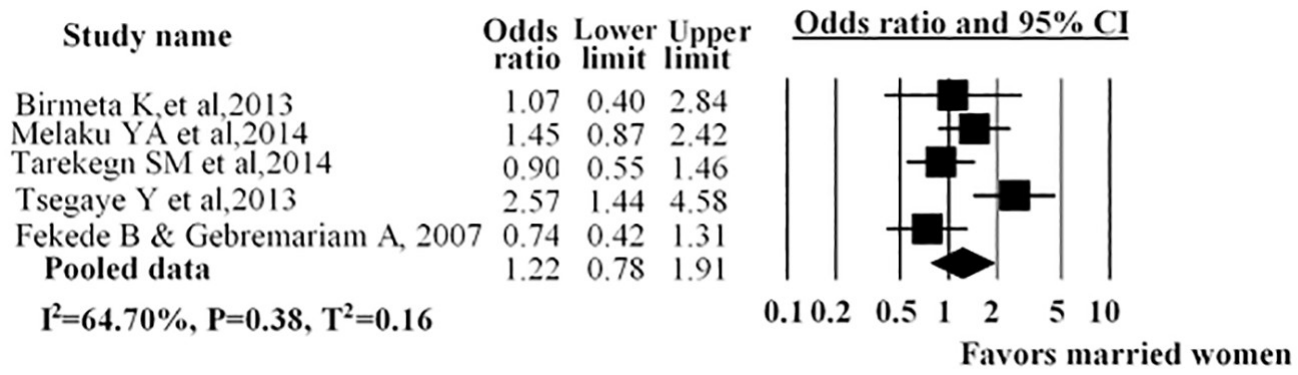
Forest plot for the association between marital status and use of antenatal care service in Ethiopia, 2016. Notes: In each study, women were assigned to marital status (Married /Other (Single, divorced, Widowed)). Each adjusted odds ratio is an estimate for a comparison between the women marriage and without marriage, with the second used as the reference category.

The association between marital status and antenatal care service utilization was described in five studies. The pooled analysis demonstrated no statistical difference among married and unmarried women (including divorced, widowed and single women) and antenatal care service utilization (AOR = 1.22; 95% CI: 0.78, 1.91) (Fig 7).

**Fig 8:**
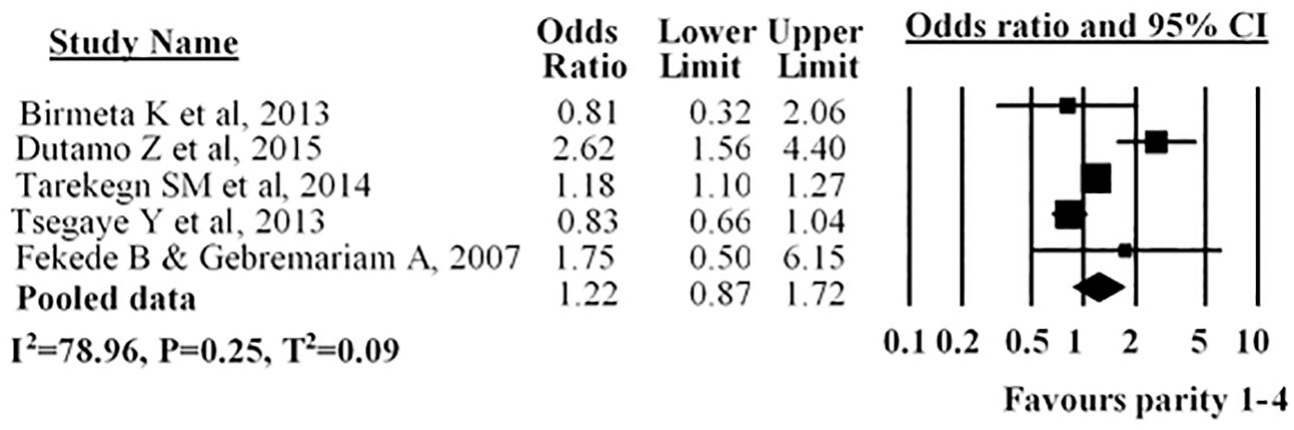
Forest plot for the association between parity and use of antenatal care service utilization in Ethiopia, 2016. Notes: In each study, women were assigned to parity (1-4 />4parity). Each adjusted odds ratio is an estimate for a comparison between the women with parity 1-4 and >4 parity, with the second used as the reference category.

Five studies described parity; three studies revealed that parity was not associated with use of antenatal care service while in two studies parity was found to have a significant effect on the use of antenatal care services. The overall pooled estimate indicated no statistical difference among women with parity of 1–4 with utilization of antenatal care compared to with women parity more than four (AOR = 1.22; 95% CI: 0.87–1.72) (Fig 8).

**Fig 9:**
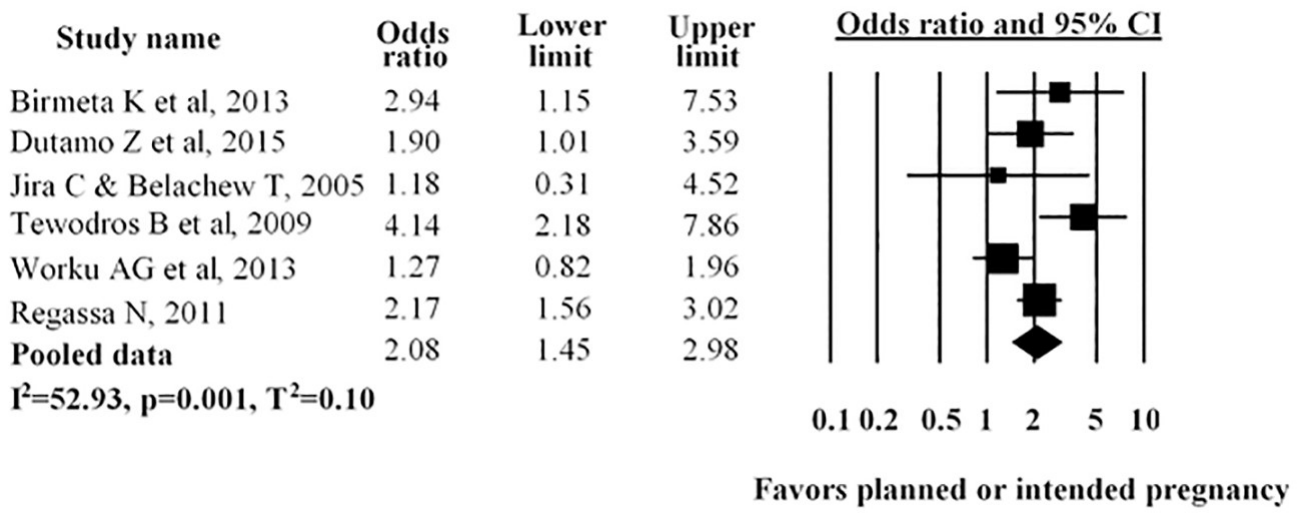
Forest plot for the association between type of pregnancy and use of antenatal care service utilization in Ethiopia, 2016. Notes: In each study, women were assigned to type of pregnancy (planned versus unplanned). Each adjusted odds ratio is an estimate for a comparison between the women with planned and unplanned pregnancy, with the second used as the reference category.

Six studies examined the impact of pregnancy intention on antenatal care service utilisation. In four studies, researchers indicated that women who planned their pregnancy were more likely to utilize antenatal care while in two studies no statistical significance was shown. The pooled odds ratio showed women who planned their pregnancy were more likely to utilize antenatal care (AOR = 2.08; 95% CI: 1.45–2.98) (Fig 9).

### Determinants of ANC which are not considered for meta-analysis

We found insufficient homogeneous classification of data to conduct meta-analyses for the following variables see S2 Table. The reasons for not conducting the meta-analysis included 1)the exposures were grouped differently across studies, 2) different reference groups and measurement of exposures were used across studies. For instance inconsistent classification of income prevented us from performing a meta-analysis of these apparent predictors of the use of antenatal care service utilization; one study described income by wealth quintile [41] while another study described by monthly income [27]. 3) the exposures were only reported in one study, or because exposure were not reported in sufficient detail to allow for data extraction.

The included studies classified occupation in different ways so we were unable to show the pooled estimates. For instance, one study classified occupation as housewife and other (civil servant, student, maid, merchant etc…) [27] and another study classified occupation as jobless or working [29]. Eight studies discussed women’s occupation with utilization of antenatal care [27–29, 37–40, 42]; four studies indicated that being a housewife had no association with the utilization of antenatal care [27, 37, 38, 42]. One study showed that women who were employed were more likely to utilize antenatal care (AOR = 1.1; 95% CI: 1.1–1.3)[29] while one study indicated that employment had no role in the utilization of antenatal care(AOR = 1.7; 95% CI: 0.8–3.3)[28]. Two studies suggested that women who were farmers or students were more likely to utilize antenatal care [38, 39]. Similarly another study revealed that women who are working in formal employment (such as in civil services) were 1.96 times more likely to use antenatal care services than their counterparts [40].

In three studies researchers found antenatal care use to be influenced by husband’s occupation [29, 30, 37]. Those women whose husbands were not employed (not working) and who were farmers were less likely to receive antenatal care [29, 30]. On the other hand, one study showed no association between antenatal care and husband’s occupation [37].

One study revealed that women in the age groups 25–34 and 35–49 are 43% and 62.6% less likely to use antenatal care services compared to women aged 15–24 [40]. Similarly one study showed women’s who are in the age group of 25–29 years were less likely to utilize antenatal care services than those 35 years and older (AOR = 0.32, 95%CI; 0.16, 0.62)[36]. Another study indicated that women aged less than or equal to 20 years at the time of first pregnancy were nearly three times more likely to use antenatal care services than women aged over 20 years at first pregnancy (AOR = 2.94, 95%CI; 1.66, 5.20)[43].

In two studies, ethnicity was shown to be significantly associated with the use of antenatal care services [29, 44] while in one study ethnicity had no statistical association with the use of antenatal care [39].

In two studies it was revealed that religion had an association with the utilization of antenatal care [36, 39] while in another four it was demonstrated that religion had no role in the utilization of antenatal care [29, 38, 40, 44].

In one study it was shown that women’s autonomy in the household was positively related to the use of antenatal care [29] while in another study researchers indicated that women’s autonomy was not associated with utilization of antenatal care [28]. In two studies it was indicated that a husband’s approval or support influences the use of antenatal care [27, 43] while in another two studies it was revealed that a husband’s approval or attitude was not statistically significant with use of antenatal care [36, 38].

Six studies assessed income in relation to antenatal care [27–29, 37, 39, 41]. In three studies researchers indicated that those women with increased family income were more likely to receive antenatal care than their counterparts [29, 37, 39] while in the remaining studies it was indicated that family income had no association with the use of antenatal care [27, 28, 41].

In four studies it was shown that having more than five living children in the family had no influence on women’s use of antenatal care [30, 36, 40, 45]. However, women who lived in a household with fewer than three children were more likely to utilize antenatal care than those living in a household size greater than five [36]. In one study researchers reported that women who had only one birth within the last five years was more likely to receive antenatal care than women who had more than one birth [29]. One study revealed that women tend to use antenatal care if their birth is the first [41]. A history of abortion was significantly associated with utilization of antenatal care [45]. In contrast another study reported that a history of abortion had no association with utilization of antenatal care service [36].

In two studies researchers indicated that use of antenatal care increased with women having awareness of the danger signs of pregnancy [27, 41] while in other two studies it was indicated that there was no statistical significance between awareness of the danger signs of pregnancy and antenatal care [28, 43]. In one study it was demonstrated that women who had a history of illness during pregnancy were more likely to use antenatal care service [43] while in another study it was stated that there was no statistical significance between complications during previous pregnancies or births and antenatal care utilization [46].

In four studies it was shown that exposure to mass media (television and radio) significantly determines utilization of antenatal care [27, 29, 37, 46]. Similarly, one study indicated that women listening to a form of mass media every day more likely to utilize antenatal care service than their counterparts [40].

In three studies it was found that knowledge or awareness of antenatal care service had a positive significant effect on the use of antenatal care service [37, 38, 44]. In one study it was revealed that previous utilization of antenatal care was a determinant of current antenatal care utilization [41]. In the other study, previous utilization had no association with the current utilization of antenatal care [46]. In two studies it was revealed that women who have an awareness of skilled care providers were more likely to receive antenatal care than their counterparts [41, 46]. In one study researchers indicated that the existence of traditional trained attendants in the area increased utilization of antenatal care service [44]. In two studies it was demonstrated that women who had awareness of places to see skilled providers for antenatal care were more likely to use antenatal care service than their counterparts [41, 46]. One study showed that women perception on the quality of the antenatal care service strongly associated with utilization of ANC [45].

In four studies researchers found that antenatal care utilization is affected by the accessibility of the services, such as availability of transport and distance to the health facilities [30, 43, 44, 46] while in three other studies it was revealed distance and availability of transport to the health facilities were not significantly associated with utilization of antenatal care [41, 45, 46].

### Publication bias

In order to check publication bias among the included studies for the meta-analysis, funnel plot and Egger’s test were carried out. Publication bias was not observed according to Egger’s test (P = 0.25) and the shape of funnel plots was symmetrical.

## Discussion

This systematic review and meta-analysis mapped out the assessment of factors affecting utilization of antenatal care among women in Ethiopia. The review highlighted the distribution, design, quality and characteristics of the studies. The review included fifteen studies from different regions. Only one study used secondary data source (Demographic and Health Survey) [29]. The remaining 14 studies collected primary data to assess antenatal care utilization. All of the included studies were community-based studies. Five studies included in this review had low quality, either because lacking information on selection of the sample population or because a sample size not justified. In the included studies, the sociodemographic factors appear to have been the most frequently studied and were among the factors most commonly associated with antenatal care use. One critical gap identified in this review was the low number (2) of longitudinal studies. In many of the studies, data were collected from women who had given birth in the last 5 years; which may be subject to recall bias.

We found that the magnitude of antenatal care service utilization was 63.77% during the period examined; this is the same as the 2016 EDHS result, which showed that the proportion of women aged 15–49 in Ethiopia who had received at least one antenatal care was 62%[18]. Antenatal care uptake was found to be lower than that found in studies in Kenya, Tanzania and Uganda [47–49]. This may be due to our inclusion criterion regarding dates of publication (2005 to 2016) while the Kenya, Tanzania and Uganda studies collected data from 2014–2015. In the included studies, the magnitude of antenatal care utilization among women ranged across regions from the highest in Oromia 85.2% and SNNPR region 66.4% to the lowest in Amhara region 32.3%. The possible explanation for this may be due to accessibility of health care service and awareness on antenatal care service utilization.

In this systematic review and meta-analysis, we found several factors that had an association with antenatal care service utilization in Ethiopia, and that there are similarities and differences between regions in factors affecting the utilization of antenatal care. The present review indicated that socio-demographic, economic, and reproductive characteristics and an awareness of the importance of antenatal care services play a significant role in the utilization of antenatal care in Ethiopia. The meta-analysis demonstrated that increased utilization of antenatal care service in Ethiopia was positively associated with urban residence, higher education among women, higher education among husbands/partners and planned pregnancy. For women aged nineteen years or younger, women who had fewer than four children and women who were married, there was no association with the utilization of antenatal care. There are notable similarities between regions regarding factors that affect antenatal care service utilization, especially maternal education and urban residence. The findings of this review are consistent with a primary study on the analysis of national survey data in seven countdown countries and a systematic review done in developing countries [24, 26, 50]. These studies revealed that residence and higher educational status was associated with uptake of antenatal care.

Empowering women through education, household wealth and increasing their decision-making power increases utilization of maternal healthcare services [51–53]. In two systematic reviews carried out in low and middle income countries, it was reported that improving women’s education increases the utilization of maternal healthcare services, including antenatal care [25, 54]. In a study in Sudan, it was revealed that lack of maternal education increased the odds of non-use of antenatal care [26]. Similar findings were found in a study carried out in Nigeria [55]. In contrast one study done in Pakistan indicated that education did not show association with utilization of antenatal care [56]. Various studies reported that women with primary or higher educational levels have a greater confidence to take actions regarding their own health and they have awareness on advantage of utilizing health services compared to women who had no education [57–59]. In addition to that, education makes women more empowered and confident by giving information on their health so they can decide to seek care during pregnancy or delivery [60].

In the systematic review, Banke-Thomas (2017) explained that urban residence is judged as a significant factor for utilization of antenatal care. In primary studies in Africa, it was also indicated that urban women used more antenatal care services than women in rural areas [61, 62]. Similarly a study conducted in Nigeria revealed that living in an urban residence increases the odds of antenatal care service utilization more than twofold [59]. This inconsistent with study done in eastern Sudan which stated that residence had no association with utilization of antenatal care [63]. The difference may be because women living in urban areas have better access to health facilities and information, and as a result they receive the services from nearby health facilities.

A systematic review on the effect of pregnancy intention on the use of antenatal care services it was shown that unintended pregnancy is associated with late initiation and inadequate use of antenatal care services [64]. Marston (2003), in a multi–country study, affirms that women experiencing unplanned pregnancies are more likely to delay antenatal care [65]. Similarly, in a study in Brazil researchers found that women having an unplanned pregnancy were less likely to utilize antenatal care than women who had planned their pregnancy [66]. Our pooled estimates showed that women whose pregnancies were planned were more likely to receive antenatal care. This is supported by studies conducted in other countries [64, 67, 68]. Similarly, in a study conducted in Kenya indicated that women who reported planned pregnancy were more likely to receive antenatal care while those who reported unplanned pregnancy were less likely to receive antenatal care [68]. It is possible that women whose pregnancies were unintended may fear the social ramifications of an unplanned pregnancy and so may avoid health services.

In this systematic review parity has no association with utilization of antenatal care. This is in line with the study conducted in Nigeria [69]. This is in contrast with the studies done in other African countries [70, 71]. These studies indicated that an increase in parity decreases the likelihood of uptake of antenatal care. The possible explanation may be women who had been pregnant many times were less motivated to go for antenatal care visits due to experience gained from previous pregnancies and births.

In the current review, we found that marital status had no association with utilization of antenatal care. Studies conducted in Rwanda and Namibia showed that women who were single, divorced or widowed were less likely to utilize antenatal care than married women [72, 73]. In contrast a study conducted in Nigeria revealed that married women were less likely to seek antenatal care services than their single counterparts [74]. One of the possible explanation mentioned in a study conducted Nigeria were married women are not economically independent and might have to seek permission from partners. The reason for married women to utilize antenatal care in Rwanda were explained by the support of their husbands.

Previous studies conducted in low-income countries demonstrated that pregnant women in the age range of 15–19 years were more likely to use antenatal care services compared to those older than 19 years [67, 72]. In this review only one study revealed that women in the age group of less than twenty years more likely to use antenatal care than women in the age group twenty and above. Our pooled estimates showed no association with a younger age and antenatal care service utilization in Ethiopia. This could be explained by the fact that a more self-confidence and gained experience from earlier pregnancies among older women; so they less likely utilize antenatal care.

Our findings suggest that both women’s and their husband’s occupation influence the utilization of antenatal care. Out of the included studies, in only one study (the analysis of the DHS data) it was revealed that women who had a job were more likely to use antenatal care. These findings may be related to both income and societal influences that come with employment outside of the home, which has also been found in other previous studies [24, 75–77]. In various studies researchers reported that utilization of antenatal care may be influenced by women’s position in the household and the approval of their husbands [78–80]. Similarly, in this review, in two studies there was an indication that the husband’s approval or support influences the use of antenatal care. A study conducted in Uganda showed that male involvement increase the use of antenatal care service utilization [81]. A possible explanation might be that autonomous women have the ability to make decisions about their own health issues, or that their husband is encouraging of their seeking medical care. This review found that there is little research on women’s autonomy in relation to antenatal care service utilization. In only one study did researchers assess women’s decision-making power to decide on healthcare spending by themselves and its influence on antenatal care service utilization.

In this review, in studies carried out in southwest and northwest Ethiopia and the national DHS analysis, it was shown that family income was positively associated with utilization of antenatal care. These findings indicate that income improves the ability to afford, and therefore access, health services [82, 83]. This is also supported by studies conducted in Sudan and Uganda [26, 84]. These findings are an important indication that affordability of health services is a key area for improving antenatal care service utilisation in Ethiopia.

In five out of fifteen studies researchers examined media exposure as a determinant variable, and in all of them it was found that exposure to mass media increases the utilization of antenatal care. Similarly a study conducted in Uganda indicated women having access to mass media at least once a week and those having access daily were more likely to use antenatal care service [85]. In a WHO report and systematic review conducted in developing countries, it was found that women with a high income and standard of living may have better access to mass media, which increases awareness of utilization of antenatal care [5, 24]. This is supported by findings from other studies implemented in low-income countries [86–88]. In these studies, it has been indicated that mass media can be an important platform to disseminate health information. A study done in Ethiopia on maternal health care service utilization revealed that access to mass media had a positive impact on uptake of maternal health care services; women living in households with high access to media have increased odds of utilizing skilled birth attendant by 24%[89].

This systematic review and meta-analysis needs careful consideration, bearing in mind some of its limitations. Firstly, more than 85% of the included studies are cross-sectional in nature, which limits our ability to assess cause-effect relationship. Secondly, there is substantial heterogeneity across the studies. The observed heterogeneity may be described by differences in the study design, the quality of the studies and sensitivity analysis. Thirdly, almost half of the included studies used women who had given birth in the five years preceding the survey as study participants, and they may have been subject to recall bias. Fourthly, we are unable to show the pooled estimates for all variables associated with antenatal care because the included studies classify the variables in different ways; for instance, women’s occupation is grouped as government employed and unemployed in one study and in other studies as employed and housewives. Despite these limitations, we were able to conduct a methodologically rigorous meta-analysis, report an adjusted odds ratio, sensitivity analysis, and sub-group analysis. The employment of a quality indictor to select only sound publications has ensured the quality of the research findings.

### Conclusion

Our review identified a number of key determinants of antenatal care use. Based on the findings of this review, empowering women, making health facilities accessible to women, increasing husband’s or partner’s participation in antenatal care, exposing women to mass media (TV and radio) and advocating antenatal care in mass media, creating ways to increase income generation by women, and providing advice on the importance of antenatal care all increased the utilization of antenatal care.

### Future research

Our review findings indicate the necessity for a range of further research: 1. Assessing the utilization of antenatal care in relation to health service barriers 2. Investigating in more detail women’s autonomy and antenatal care service utilization 3. Examining the utilization of antenatal care at different levels (individual and community) by using qualitative methods.

## Supporting information

S1 Table Search method used in Medline. (DOCX)

S2 Table Data collected from the included studies. (DOCX)

S1 File Excluded articles and reason. (DOCX)

S2 File completed PRISMA Checklist for ANC new. (DOC)

S3 File Completed MOOSE checklist. (DOC)

## Abbreviations

ANC: Antenatal care
EDHS: Ethiopian Demographic and Health Survey
NOS: Newcastle-Ottawa scale
