# Supplementary material for: Factors affecting utilization of antenatal care in Ethiopia: A systematic review and meta-analysis
Source: PLoS One. 2019 Apr 11;14(4):e0214848. doi: 10.1371/journal.pone.0214848 (PMC6459485; doi:10.1371/journal.pone.0214848)
Supplement: S1 Table — (DOCX) [file pone.0214848.s001.docx]

Search method used in Medline: - limiting English language and study conducted between 2002 to 2016

1. Antenatal care --- 5278
2. Prenatal care ------ 26421
3. Maternal health care – 11715
4. 1 or 2 or 3------ 38786
5. Utilization ------ 146494
6. Determinants - 115101
7. Factors affecting --- 32349
8. 4 and 5------ 1380
9. 4 or 6 ---------152986
10. 4 and 7--------191
11. Ethio?--------- 8508
12. 8 or 9 ----------152986
13. 9 and 10 -------191
14. 12 and 13 ------191
15. 4 or 5 or 6 or 7—327106
16. 11 and 15 ---------751
17. 8 or 9 or 10 -------152986
18. 11 and 17 ---------537
19. 4 and 11------------330
20. 18 or 19 ------------537
21. 18 and 19 ----------330
22. 20 and 21 ----------330
