# Supplementary material for: Factors affecting utilization of antenatal care in Ethiopia: A systematic review and meta-analysis
Source: PLoS One. 2019 Apr 11;14(4):e0214848. doi: 10.1371/journal.pone.0214848 (PMC6459485; doi:10.1371/journal.pone.0214848)
Supplement: S1 File — (DOCX) [file pone.0214848.s003.docx]

**Excluded articles**

The following 31 articles were excluded after reviewing the full texts because of exposure and outcome definition, timing of antenatal care, initiation of antenatal care, quality of antenatal care service utilization etc consider risk group and insufficient data. After reading the full text based on eligibility criteria some of articles excluded with the following reason

- The data collected already form antenatal care service user
- Focusing on one factors without clear description
- Error in result part i.e the magnitude in the abstract part is different from the main result part
- Includes the 2000 EDHS result which not include the FAC and included women who received the ANC from HEWs
- Problem in the analysis particularly no scoring methods mentioned in the methods part for knowledge question.
- The result comparing the residence
- The dependent variable is attendance of four ANC visits
- Comparing by demographic centres
- It is focusing only in rural women and done by other author (which is included in the study)- EDHS 2011
- Focusing on general health care, number of visits and unable to get full text and the data already analysed by Tarekegne
- The magnitude is not consistent with the EDHS 2011. Not adjusted for confounder.

| S.No | Study | Reason for exclusion |
| --- | --- | --- |
| 1 | Biratu BT, Lindstrom DP. The influence of husbands' approval on women's use of prenatal care: Results from Yirgalem and Jimma towns, south west Ethiopia. Ethiopian Journal of Health Development. 2006;20(2):84-92. | The data collected before 2002(Not include the FANC) |
| 2 | Ejigu T, Woldie M, Kifle Y. Quality of antenatal care services at public health facilities of Bahir-Dar special zone, Northwest Ethiopia. BMC Health Serv Res. 2013;13:443. | Focus on quality of antenatal care |
| 3 | Gedefaw M, Muche B, Aychiluhem M. Current Status of Antenatal Care Utilization in the Context of Data Conflict: The Case of Dembecha District, Northwest Ethiopia. Open Journal of Epidemiology. 2014;04(04):208-16. | Problem in the findings |
| 4 | Gela BD. Antenatal Care Utilization and Associated Factors from Rural Health Extension Workers in Abuna Gindeberet District, West Shewa, Oromiya Region, Ethiopia. American Journal of Health Research. 2014;2(4):113. | Include ANC user only from Health extension worker |
| 5 | Gudayu TW, Woldeyohannes SM, Abdo AA. Timing and factors associated with first antenatal care booking among pregnant mothers in Gondar Town; North West Ethiopia. BMC Pregnancy Childbirth. 2014;14:287. | The study participants were antenatal care user |
| 6 | Karim AM, Admassu K, Schellenberg J, Alemu H, Getachew N, Ameha A, et al. Effect of Ethiopia’s health extension program on maternal and newborn health care practices in 101 rural districts: a dose-response study. PLoS ONE [Electronic Resource]. 2013;8(6):e65160. | Focus on health care practice |
| 7 | Little A, Medhanyie A, Yebyo H, Spigt M, Dinant GJ, Blanco R. Meeting community health worker needs for maternal health care service delivery using appropriate mobile technologies in Ethiopia.[Erratum appears in PLoS One. 2014;9(1). | technical needs of Health Extension Workers (HEWs) and midwives for maternal health |
| 8 | Materia E, Mehari W, Mele A, Rosmini F, Stazi M, Damen H, et al. A community survey on maternal and child health services utilization in rural Ethiopia. European journal of epidemiology. 1993;9(5):511-6. | Data collected before 2002 |
| 10 | Medhanyie A, Spigt M, Kifle Y, Schaay N, Sanders D, Blanco R, et al. The role of health extension workers in improving utilization of maternal health services in rural areas in Ethiopia: a cross sectional study. BMC Health Services Research. 2012;12:352. | This study focuses on the extent to which these trained community health workers have contributed to the improvement of utilization of maternal health services |
| 11 | Mekonnen Y, Mekonnen A. Factors Influencing the Use of Maternal Healthcare Services in Ethiopia. Journal of health, population, and nutrition. 2003;21(4):374-82. | Old data (collected before 2002) |
| 12 | Nigatu D, Gebremariam A, Abera M, Setegn T, Deribe K. Factors associated with women's autonomy regarding maternal and child health care utilization in Bale Zone: a community based cross-sectional study. BMC Womens Health. 2014;14:79. | The dependent variable were women’s autonomy and focus on knowledge of MCH |
| 13 | Tekelab T, Berhanu B. Factors Associated with Late Initiation of Antenatal Care among Pregnant Women Attending Antenatal Clinic at Public Health Centers in Kembata Tembaro Zone, Southern Ethiopia. Science, Technology and Arts Research Journal. 2014;3(1):108. | The aim of the study was late initiation of antenatal care |
| 14 | Wado YD, Afework MF, Hindin MJ. Unintended pregnancies and the use of maternal health services in Southwestern Ethiopia. BMC International Health & Human Rights. 2013;13:36. | The study participants are different from our inclusion criteria |
| 15 | Wakbulcho M, Möller B. Attitudes toward current pregnancy among women attending an antenatal clinic in Ethiopia. International Journal of Gynecology & Obstetrics. 1994;46(1):61-2. | The study examine attitude on pregnancy |
| 16 | Woldemicael G, Tenkorang EY. Women's autonomy and maternal health-seeking behavior in Ethiopia. Matern Child Health J. 2010;14(6):988-98. | The aim was on health seeking behaviour |
| 17 | Worku AG, Yalew AW, Afework MF. Availability and components of maternity services according to providers and users perspectives in North Gondar, Northwest Ethiopia. Reproductive Health. 2013;10:43. | Assess the availability and the components of maternity services according to the perspectives of service users and providers. |
| 18 | Alemayehu T, Haidar J, Habte D. Utilization of antenatal care services among teenagers in Ethiopia: A cross sectional study. Ethiop. J. Health Dev. 2010;24(3):221-225 | Data collected before 2002 |
| 19 | Chemir et al.: Satisfaction with focused antenatal care service and associated factors among pregnant women attending focused antenatal care at health centers in Jimma town, Jimma zone, South West Ethiopia; a facility based cross-sectional study triangulated with qualitative study. BMC Research Notes 2014 7:164. | On satisfaction of antenatal care |
| 20 | Zegeye AM, Bitew BD, Koye DN. Prevalence and Determinants of Early Antenatal Care Visit among Pregnant Women Attending Antenatal Care in Debre Berhan Health Institutions, Central Ethiopia. Afr J Reprod Health 2013; 17[4]: 130-136 | Focus on early antenatal care user. |
| 21 | Woldemicael G. Do women with higher autonomy seek more maternal and child health-care? Evidence from Ethiopia and Eritrea | Mixed result from two countries |
| 22 | Muleta M, Gerrits T, Both R. Husbands’ Roles in Prenatal Care in Addis Ababa. (unpublished thesis) | Use only qualitative methods and ANC user and non-user not mentioned clearly. |
| 23 | Gudayu T. Proportion and Factors Associated with late Antenatal Care Booking among Pregnant Mothers in Gondar Town, North West Ethiopia. Afr J Reprod Health 2015; 19[2]: 94-100 | Describe only about late initiation of ANC |
| 24 | Sekata D. Modeling the Number of Antenatal Care Service Visits Among Pregnant Women in Rural Ethiopia: Zero Inflated and Hurdle Model Specifications. International Journal of Healthcare Sciences 2015. 3( 1). | The result not clearly indicated and the analysis already done by Tarekegn SM et al, 2014 |
| 25 | Mekonnen Y, Mekonnen A. Utilization of Maternal Health Care Services in Ethiopia. Calverton, Maryland, USA: ORC Macro.2002 | The data collected before 2002 |
| 26 | Terye ND. Multilevel Modeling of Utilization of Maternal Health Care Services in Ethiopia. Ethiopian e-journal for res and inn.2015;7(1) | Duplicate and statistical problem. The 2011 EDHS alredy analysed by Tarekegn SM et al, 2014 |
| 27 | Aliy J, Hailemariam D. Determinants of equity in utilization of maternal health services in Butajira, Southern Ethiopia .Ethiop. J. Health Dev. 2012;26 Special Issue 1:265-270 | The objective not specified and ANC user not indicated. |
| 28 | Endashaw T, Fantahun M. Assessment of late entry to antenatal care and its predictors among ANC attendees in Gambella region.June2010(Unpublished) | The study participants were ANC user. |
| 29 | Getachew T, AbajobirAA,Aychiluhim M. Focused Antenatal Care Service Utilization and Associated Factors in Dejen and Aneded Districts, Northwest Ethiopia. Primary Health Care 2014 4:170. | The aim was on the number of visits rather than ANC utilization |
| 30 | Berhe KK , Welearegay HG , AberaGB , Kahsay HB, Kahsay AB. Assessment of Antenatal Care Utilization and its Associated Factors Among 15 to 49 Years of Age Women in Ayder Kebelle, Mekelle City 2012/2013; A Cross Sectional Study. American Journal of Advanced Drug Delivery | The result is not consistent. The finding in the abstract part indicated different from the main findings. |
| 31 | Yilala S eta al. Assessment of late initiation of antenatal care and associated factors among antenatal care attendees in selected health centers of Addis Ababa, Ethiopia, 2015(Unpublished) | The outcome was late initiation of antenatal care. |
|  |  |  |
